# Effect of Comparable Carbon Chain Length Short- and Branched-Chain Fatty Acids on Adipokine Secretion from Normoxic and Hypoxic Lipopolysaccharide-Stimulated 3T3-L1 Adipocytes

**DOI:** 10.3390/biomedicines12112621

**Published:** 2024-11-16

**Authors:** Ala Alzubi, Jennifer M. Monk

**Affiliations:** Department of Human Health and Nutritional Sciences, University of Guelph, Guelph, ON N1G 2W1, Canada; aalzubi@uoguelph.ca

**Keywords:** short-chain fatty acids, branched-chain fatty acids, obesity, hypoxia, butyrate, inflammation, adipose tissue, adipocyte

## Abstract

**Background:** Microbial fermentation of non-digestible carbohydrates and/or protein produces short-chain fatty acids (SCFA), whereas branched-chain fatty acids (BCFA) are produced from protein fermentation. The effects of individual SCFA and BCFA of comparable carbon chain length on adipocyte inflammation have not been investigated. **Objective**: To compare the effects of SCFA and BCFA on inflammatory mediator secretion in an adipocyte cell culture model designed to recapitulate obesity-associated adipocyte inflammation under normoxic and hypoxic conditions. **Methods:** The 3T3-L1 adipocytes were cultured (24 h) without (Control, Con) and with 1 mmol/L of SCFA (butyric acid (But) or valeric acid (Val)) or 1 mmol/L of BCFA (isobutyric acid (IsoBut) or isovaleric acid (IsoVal)) and were unstimulated (cells alone, *n* = 6/treatment), or stimulated with 10 ng/mL lipopolysaccharide (LPS, inflammatory stimulus, *n* = 8/treatment) or 10 ng/mL LPS + 100 µmol/L of the hypoxia memetic cobalt chloride (LPS/CC, inflammatory/hypoxic stimulus, *n* = 8/treatment). **Results**: Compared to Con + LPS, But + LPS reduced secreted protein levels of interleukin (IL)-1β, IL-6, macrophage chemoattractant protein (MCP)-1/chemokine ligand (CCL)2, MCP3/CCL7, macrophage inflammatory protein (MIP)-1α/CCL3 and regulated upon activation, normal T cell expressed, and secreted (RANTES)/CCL5 and decreased intracellular protein expression of the ratio of phosphorylated to total signal transducer and activator of transcription 3 (STAT3) and nuclear factor kappa B (NFκB) p65 (*p* < 0.05). Val + LPS reduced IL-6 secretion and increased MCP-1/CCL2 secretion compared to Con + LPS and exhibited a different inflammatory mediator secretory profile from But + LPS (*p* < 0.05), indicating that individual SCFA exert individual effects. There were no differences in the secretory profile of the BCFA IsoBut + LPS and IsoVal + LPS (*p* > 0.05). Alternatively, under inflammatory hypoxic conditions (LPS/CC) Val, IsoVal, and IsoBut all increased secretion of IL-6, MCP-1/CCL2 and MIP-1α/CCL3 compared to Con (*p* < 0.05), whereas mediator secretion did not differ between But and Con (*p* > 0.05), indicating that the proinflammatory effects of SCFA and BCFA was attenuated by But. Interestingly, But + LPS/CC decreased STAT3 activation versus Con + LPS/CC (*p* < 0.05). **Conclusions**: The decreased secretion of inflammatory mediators that is attributable to But highlights the fact that individual SCFA and BCFA exert differential effects on adipocyte inflammation under normoxic and hypoxic conditions.

## 1. Introduction

Obesity prevalence is increasing and is associated with a low-grade chronic inflammatory state that promotes metabolic dysfunction including insulin resistance and dyslipidemia [1,2,3,4,5]. Adipose tissue hypoxia further exacerbates the production of inflammatory mediators (i.e., adipokines) and subsequent metabolic dysfunction, leading to a more severe obese phenotype [5,6,7,8,9,10]. Identifying dietary components that can beneficially modulate the inflammatory adipokine profile and improve adipocyte or adipose tissue metabolic function represents a non-invasive obesity intervention approach.

Microbial fermentation products from non-digestible dietary components represent one important source of biologically active metabolites that can affect host physiological function locally (i.e., within the gastrointestinal epithelium) and systemically (i.e., extra-intestinally) by translocating across the intestinal epithelial barrier to reach the systemic circulation [11,12]. Short-chain fatty acids (SCFA) are predominantly produced from microbial fermentation of non-digestible carbohydrates (NDC) and to a lesser degree from the fermentation of undigested proteins [13]. Biochemically, SCFA can be in the acid form (e.g., acetic acid) or the salt or ester form (e.g., acetate) and differ in their carbon number, including acetate (two carbons), propionate (three carbons), butyrate (four carbons), valerate (five carbons), etc. The amount of each SCFA produced is dependent upon the amount and fermentability of the dietary substrate consumed, the composition and activity of SCFA-producing microbial species in the microbiome, wherein species can be non-fermenters or they can differ in the amount and type of different SCFA that will be produced [14,15,16,17]. Therefore, a range of SCFA concentrations are reported wherein systemic concentrations are considerably lower compared to fecal levels [18,19,20,21,22,23,24,25,26]. In extra-intestinal tissues such as adipose tissue, skeletal muscle, liver, and the pancreas, SCFA have been shown to modulate host physiological function by exerting metabolic effects [6,10,13,27,28,29,30,31,32]. In adipose tissue or adipocytes, SCFA have been shown to decrease lipolysis and increase triglyceride accumulation, along with increasing glucose uptake and adipogenesis [33,34,35,36,37,38,39]. SCFAs of differing chain lengths are frequently assumed to function in the same manner, as they have varying binding affinities for shared receptors, the G protein-coupled receptors (GPCR), mainly GPR41, GPR43, and GPR109a [40]. However, individual SCFAs of varying carbon chain lengths can exert differential effects and cannot be assumed to function similarly, as shown previously with inflammatory and metabolic function in skeletal muscle cells [41,42]. Many of the metabolic effects of SCFA investigated in adipose have focussed on select individual SCFA, such as only acetate [34,35,38]; only butyrate [33]; or studies assessing differing outcomes between acetate, propionate, and butyrate [32,43,44], leaving longer-chain lengths, such as valerate [39], largely understudied.

Apart from metabolic effects, the influence of different SCFA on adipokine production and/or secretion has been studied in a limited manner, with the majority of studies focussed specifically on leptin and adiponectin, leaving an array of cytokines and chemokines that influence adipose tissue function under-investigated. Stimulation of leptin secretion by propionate, butyrate, and valerate has been reported in mouse adipose tissue cultures, wherein acetate had no effect [44], thereby demonstrating the capacity for individual SCFA to differentially affect adipose tissue adipokine secretion. Leptin gene expression in primary adipocytes from obese donors was increased by a 1 mmol/L treatment of acetate, propionate, and butyrate, with no effect on adiponectin gene expression [37]. Interestingly, in the same study primary adipocytes isolated from obese donors with type 2 diabetes responded differently to the same dose of each SCFA, wherein gene expression of both leptin and adiponectin were increased by each SCFA [37]. Yet, in another study adiponectin secretion from primary brown adipose tissue and white adipose tissue exhibited increased adiponectin secretion in response to 10 mmol/L acetate treatment, wherein leptin secretion was unaffected [45]. Collectively, these results highlight the conflicting findings of different SCFAs, particularly within the context of differing adipose tissue experimental conditions. This highlights the need for assessment of SCFA effects within the context of experimental conditions that mimic distinct elements of the AT tissue physiological state and the need for appropriately crafted cell culture models to recapitulate the critical and nuanced features of obese adipose tissue. Little is known about the other inflammatory mediators, namely cytokines and chemokines, that are produced from obese adipose tissue, apart from a reduction in the inflammatory cytokine (tumor necrosis factor (TNF)α) and chemokines (regulated upon activation, normal T cell expressed, and secreted (RANTES)/chemokine ligand (CCL)5, macrophage inflammatory protein(MIP)-1β/CCL4 and MIP-1α/CCL3) following the culture of obese human adipose tissue with 3 mM of propionate [46]. This highlights the need for a further study assessing the effects of SCFAs with longer chain lengths on inflammatory mediator secretion using cell culture models that recapitulate obese adipocyte critical features.

Branched-chain fatty acids (BCFA) are found directly in dairy or ruminant dietary sources typically in forms with longer carbon chain lengths [47] and are produced by the microbiome through protein fermentation, specifically the fermentation of branched-chain amino acids [48]. Additionally, increased levels of isobutyric acid have been reported following ingestion of some dietary fibres such as polydextrose [49]. BCFA have the distinguishing feature of a side chain or methyl branches connected to the carbon backbone [50]. Longer chain length BCFA exist, in addition to the short BCFA that are analogous in carbon chain number to SCFA, for example, isobutyric acid/isobutyrate would be the analogous BCFA to the SCFA butyric acid/butyrate. Microbial synthesis of BCFA, such as isobutyrate and isovalerate are considerably lower compared to SCFA production and have been reported at 1.68–2.43 mM [48]. Little is known about the effects of BCFA in adipose tissue; however, adipose tissue levels of monomethyl branched-chain fatty acids with longer carbon chain lengths decrease in obesity and subsequently increase in response to weight loss, wherein their adipose tissue levels are positively correlated with skeletal muscle insulin-stimulated glucose uptake [51]. Similarly, these longer carbon chain length BCFA dose-dependently modulated the expression of lipid metabolism genes in human adipose tissue; however, they exerted differential effects on fatty acid synthase and interleukin (IL)-6 expression, highlighting unique effects attributable to different BCFA that could attenuate or exacerbate obesity-related metabolic dysfunction [52]. Shorter carbon chain length BCFAs, namely isobutyric acid and isovaleric acid, have been shown to decrease lipogenesis, inhibit lipolysis, and decrease phosphorylation of hormone-sensitive lipase in adipocytes [53]. Furthermore, both BCFAs increased glucose uptake, wherein isobutyric acid had the strongest effect on both basal and insulin-stimulated glucose uptake, whereas the effects of isovaleric acid were observed at a high concentration under basal stimulation conditions [53]. Collectively, these data demonstrate the ability of short carbon chain length BCFAs to regulate adipose tissue energy homeostasis; however, their effects on adipocyte adipokine production remain unknown. Therefore, the objective of this study was to determine the effects of analogous carbon chain length SCFA and BCFA on adipocyte inflammatory mediator secretion in three different states, namely: (i) unstimulated normoxic conditions, (ii) inflammatory normoxic conditions, and (iii) inflammatory hypoxic conditions, which are reflective of an obese adipocyte microenvironment.

## 2. Materials and Methods

### 2.1. Cell Culture Conditions and Differentiation

Cell culture conditions for 3T3-L1 murine pre-adipocytes were performed according to the manufacturer’s instructions (CL-173; American Type Culture Collection, Manassas, VA, USA). In brief, the 3T3-L1 pre-adipocytes were grown in basic Dulbecco’s modified Eagle’s medium (DMEM, #SH30022.01; HyClone, Logan, UT, USA) supplemented with 4 mmol/L L-glutamine, 4500 mg/L glucose 10% (*v*/*v*) low endotoxin sterile-filtered fetal bovine serum (FBS; #F2442; Millipore-Sigma, Oakville, ON, Canada) and 1% (*v*/*v*) penicillin-streptomycin (#15140122; Fisher Scientific, Mississauga, ON, Canada) in a humidified incubator at 37 °C with 5% carbon dioxide, as described [54]. Pre-adipocytes were seeded in 6-well plates, differentiated 2 days post-confluence (day 0) with basic DMEM containing 1 µmol/L dexamethasone (#D4902), 0.5 mmol/L 3-isobutyl-1-methylxanthine (#I5879) and 10 µg/mL insulin (#I9278) (all from Millipore-Sigma), and then matured in DMEM supplemented with 10 µg/mL insulin until day 8, when they were ready for use in experiments, as described [54]. Media was changed every two days. On day 8 post-differentiation, adipocytes were incubated for 12 h in serum-free DMEM containing 1% (*v*/*v*) penicillin-streptomycin prior to the addition of the experimental treatments.

### 2.2. Experimental Treatment Conditions 

Cells were treated for 24 h with media alone (control, Con) or with media containing 1 mmol/L of the SCFA sodium butyric acid (But; #W222119; Millipore-Sigma) or valeric acid (Val; #W310107, Millipore-Sigma), or 1 mmol/L of the BCFA isobutyric acid (Iso-But; #W222208; Millipore-Sigma) or isovaleric acid (Iso-Val; #W310204; Millipore-Sigma), concentrations that have been used previously [33,36,37,39,53,55]. These experimental treatment groups were stimulated with three separate stimulation conditions: (i) unstimulated [negative control, *n* = 6/experimental group: cells alone with media (Con) or media plus each SCFA or BCFA alone (But, Val, IsoBut or IsoVal)]; (ii) an inflammatory stimulus of 10 ng/mL lipopolysaccharide [LPS, from *Escherichia coli* 055:B5 (#L5418; Millipore-Sigma); *n* = 8–9/experimental group: Con + LPS, But + LPS, Val + LPS, IsoBut + LPS, IsoVal + LPS]; or (iii) a combined inflammatory and hypoxic stimulus of 10 ng/mL LPS plus 100 µmol/L of the hypoxia-mimetic compound, cobalt chloride [(CC; #15862; Millipore-Sigma); LPS/CC, *n* = 8–9/experimental group: Con + LPS/CC, But + LPS/CC, Val + LPS/CC, IsoBut + LPS/CC, IsoVal + LPS/CC]. The dose of LPS utilized in these experiments recapitulates the circulating endotoxin levels reported in obese humans [56] and rodent high-fat diet-induced obesity models [57,58]. The dose of CC was used to mimic the effects of low (1%) oxygen tension between adipocytes over 24 h without affecting cell viability [59,60]. Cell viability in response to all treatment conditions was assessed using Trypan blue exclusion and exceeded 90%. Furthermore, there was no difference in total cellular protein content measured in LPS/CC-treated cell cultures compared to unstimulated, as seen previously using the same LPS/CC stimulation conditions [60]. After 24 h, culture supernatant was collected and stored at −80 °C to await secreted adipokine analysis, and cells were lysed using the lysis buffer from the RNA/Protein Purification Plus Kit (#48200; Norgen Biotek Corp., Thorold, ON, Canada), which was collected and stored at −80 °C to await RNA and cellular protein isolation using the same kit, as per the manufacturer’s instructions.

### 2.3. Gene Expression Analysis

Isolated RNA was quantified using the Nanodrop UV spectrophotometer (Model ND-2000; Thermo-Fisher Scientific, Burlington, ON, Canada). A total of 2 µg of cDNA was synthesized using the high-capacity reverse transcription kit (Applied Biosystems, Forest City, CA, USA). Real-time PCR was conducted using the CFX q-PCR system (Bio-Rad, Mississauga, ON, Canada), as previously described [61]. Primer sequences (Table 1) for the hypoxia-sensitive target genes hypoxia-inducible factor-1α (*Hif1α*) and angiopoietin-like protein 4/fasting-induced adipose factor (*Angptl4*) [7,62] and the housekeeping gene, ribosomal protein, large, P0 (*Rplp0*) have been published previously [60]. Target gene expression was normalized to the housekeeping gene *Rplpo* and relative differences in gene expression between treatment groups were determined using the ΔΔCT method in comparison to unstimulated adipocytes and are expressed as fold-changes.

### 2.4. Secreted Protein Analysis

Secreted cytokines (IL-1β, IL-6, IL-10, TNFα) chemokines (monocyte chemoattractant protein (MCP)-1/CCL2, MCP-3/CCL7, MIP-1α/CCL3, MIP-1β/CCL4 and RANTES)/CCL5) were simultaneously measured in culture supernatant using the mouse Bio-Plex Pro kit (Bio-Rad) as per the manufacturer’s instructions using the Bio-Plex 200 system and Bio-Plex Manager software, version 6.0 (Bio-Rad). IL-10 was below the assay limit of detection in both unstimulated, LPS-stimulated, and LPS/CC-stimulated conditions for all treatment groups. Similarly, secreted proteins for all inflammatory mediators were below the limit of detection for the unstimulated culture condition for each experimental treatment group (Con, But, Val, IsoVal, and IsoBut).

### 2.5. Intracellular Protein Analysis

Total intracellular protein was quantified using the bicinchoninic assay according to the manufacturer’s instructions (#PI23225; Thermo-Fisher Scientific). An equal amount of protein (10 µg/sample/assay) was used to measure the ratio of phosphorylated to total expression of transcription factors STAT3 (p-STAT3 [Y705]: Total STAT3; #85-86102-11 and #85-86101-11, respectively) and NFκB p65 (p-NFκB p65 [Ser536]: Total NFκB p65; #85-86082-11 and #85-86081-11, respectively) using enzyme-linked immunosorbent assay, as per the manufacturer’s instructions (Thermo-Fisher Scientific). The final absorbance was measured at 450 nm using SpectraMax M5e Multimode Plate Reader (Molecular Devices, San Jose, CA, USA).

### 2.6. Statistical Analysis

All data are expressed as means ± SEM. Data were analyzed by one-way ANOVA followed by Tukey’s multiple comparisons test for post-hoc analysis between experimental treatment groups (*p* ≤ 0.05). The Shapiro–Wilk test was used to test for normality. All analyses were conducted using GraphPad Prism, version 10 (GraphPad Software, Inc., La Jolla, CA, USA).

## 3. Results

### 3.1. Effect of SCFA and BCFA on Secreted Inflammatory Mediators in LPS-Stimulated Adipocytes

**Figure 1:**
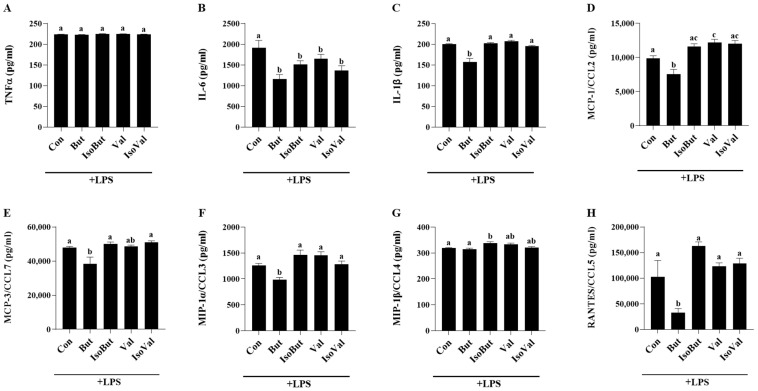
Effect of individual SCFA and BCFA (1 mmol/L) on secreted protein levels of inflammatory mediators (**A**) TNFα, (**B**) IL-6, (**C**) IL-1β, (**D**) MCP-1/CCL2, (**E**) MCP-3/CCL7, (**F**) MIP-1α/CCL3, (**G**) MIP-1β/CCL4, and (**H**) RANTES/CCL5 in response to 24 h LPS (10 ng/mL) stimulation in 3T3-L1 adipocytes. Bars represent mean values ± SEM (*n* = 8–9/experimental group). Data were analyzed by one-way ANOVA followed by Tukey’s multiple comparison test. Bars not sharing a lower-case letter differ (*p* < 0.05).

In the unstimulated condition, all cytokine and chemokine inflammatory mediators assessed in this study were below the limit of detection in each experimental group (Con, But, Val, IsoBut, and Isoval), thereby indicating that the presence of either SCFA (But or Val) or BCFA (IsoBut or IsoVal) alone did not elicit an inflammatory response from adipocytes. Conversely, inflammatory mediator secretion in response to LPS stimulation was detectable and differed between Con and the SCFA and BCFA treatments, as shown in Figure 1. There was no difference in secreted TNFα levels between experimental groups (*p* > 0.05; Figure 1A). Both the SCFA treatments (But and Val) and BCFA treatments (IsoBut and IsoVal) reduced IL-6 secretion in comparison to Con (*p* < 0.05; Figure 1B), but did not differ from each other. But + LPS also reduced adipocyte secretion of other inflammatory mediators including IL-1β, MCP-1/CCL2, MIP-1α/CCL3, and RANTES/CCL5 compared to Con + LPS, and the other SCFA and BCFA treatment groups (*p* < 0.05; Figure 1C,D,F,H). But + LPS reduced MCP-3/CCL7 secretion compared to both Con + LPS and IsoBut + LPS (*p* < 0.05; Figure 1E), however, MCP3/CCL7 secretion did not differ between the SCFA treatment groups (But + LPS and Val + LPS; *p* > 0.05). Conversely, Val + LPS increased the secretion of MCP-1/CCL2 compared to both Con + LPS and But + LPS (*p* < 0.05; Figure 1D), but secretion did not differ from the comparable BCFA, IsoVal + LPS (*p* > 0.05). There were no other differences in inflammatory mediator secretion in response to LPS stimulation between Val and IsoVal (*p* > 0.05). The BCFA, IsoBut + LPS, and IsoVal + LPS did not differ for any inflammatory mediators assessed; however, IsoBut + LPS increased secretion of MIP-1β compared to both Con + LPS and But + LPS (Figure 1G).

### 3.2. Effect of SCFA and BCFA on Transcription Factor Activtation in LPS-Stimulated Adipocytes

**Figure 2:**
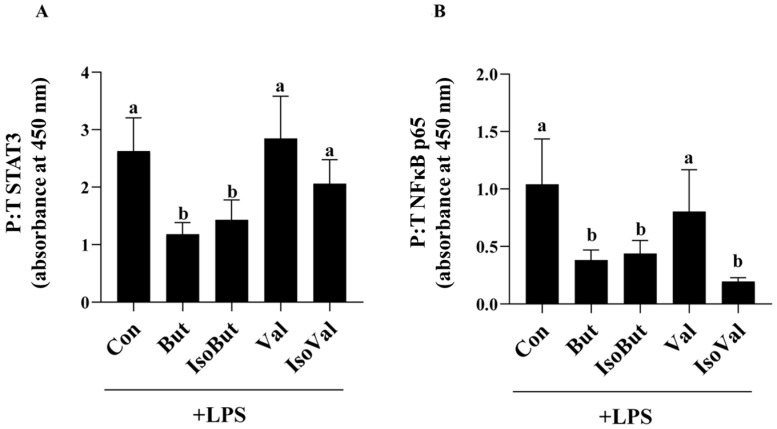
Effect of individual SCFA and BCFA (1 mmol/L) on (**A**) STAT3, and (**B**) NFκB p65 cellular protein expression (ratio of phosphorylated to total, P:T) in response to 24 h LPS (10 ng/mL) stimulation in 3T3-L1 adipocytes. Bars represent mean values ± SEM (*n* = 8–9/experimental group). Data were analyzed by one-way ANOVA followed by Tukey’s multiple comparison test. Bars not sharing a lower-case letter differ (*p* < 0.05).

In response to LPS stimulation, only But and IsoBut reduced the ratio of phosphorylated to total STAT3 intracellular protein levels compared to Con, Val, and IsoVal (*p* < 0.05; Figure 2A). There was no difference in phosphorylated to total STAT3 protein levels between Con, Val, and IsoVal (*p* > 0.05). Similarly, But, IsoBut, and Val reduced the ratio of phosphorylated to total NFκB p65 compared to Con and IsoVal in response to LPS stimulation (*p* < 0.05; Figure 2B).

### 3.3. Effect of SCFA and BCFA on the Expression of Hypoxia-Sensitive Genes in Combined LPS/CC-Stimulated Adipocytes

**Figure 3:**
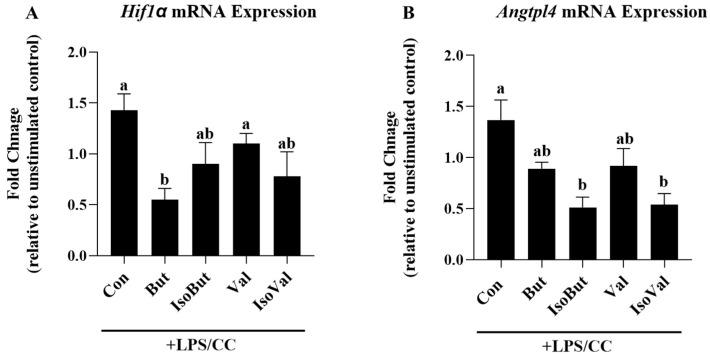
Effect of individual SCFA and BCFA (1 mmol/L) on mRNA expression of hypoxia-sensitive genes (**A**) *Hif1α*, and (**B**) *Angtpl4* in response to 24 h combined inflammatory and hypoxic stimulation conditions (LPS/CC; 10 ng/mL LPS + 100 µmol/L CC) in 3T3-L1 adipocytes. Bars represent mean values ± SEM (*n* = 8–9/experimental group) and data are presented as fold-changes compared to unstimulated control (adipocytes alone). Data were analyzed by one-way ANOVA followed by Tukey’s multiple comparison test. Bars not sharing a lower-case letter differ (*p* < 0.05).

Expression of the hypoxia-sensitive genes *Hif1α* and *Angptl4* in LPS/CC-stimulated adipocyte cultures is shown in Figure 3. Expression of *Hif1α* and *Angptl4* were increased between the unstimulated and LPS/CC-stimulated experimental conditions. Under combined inflammatory and hypoxic experimental conditions *Hif1α* mRNA expression was decreased by But + LPS/CC compared to Con + LPS/CC and Val + LPS/CC (*p* < 0.05; Figure 3A), but did not differ from IsoBut + LPS/CC and IsoVal + LPS/CC (*p* > 0.05). There was no difference in *Hif1α* mRNA expression between any other experimental groups. Alternatively, both IsoBut + LPS/CC and IsoVal + LPS/CC decreased *Angtpl4* mRNA expression compared to Con + LPS/CC (*p* < 0.05; Figure 3B). There was no difference in *Angptl4* mRNA expression between individual SCFA (But versus Val) or BCFA (IsoBut versus IsoVal) or between comparable carbon chain length SCFA and BCFA (i.e., But versus IsoBut or Val versus IsoVal) (*p* > 0.05; Figure 3B).

### 3.4. Effect of SCFA and BCFA on the Secretion of Inflammatory Mediators in LPS/CC-Stimulated Adipocytes

**Figure 4:**
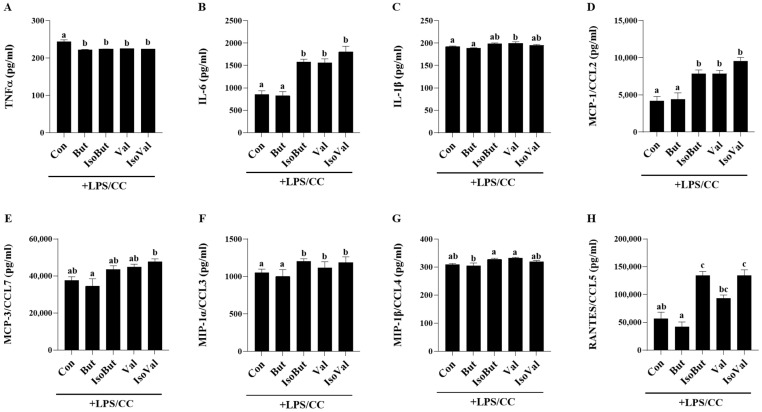
Effect of individual SCFA and BCFA (1 mmol/L) on secreted protein levels of inflammatory mediators (**A**) TNFα, (**B**) IL-6, (**C**) IL-1β, (**D**) MCP-1/CCL2, (**E**) MCP-3/CCL7, (**F**) MIP-1α/CCL3, (**G**) MIP-1β/CCL4, and (**H**) RANTES/CCL5 in response to 24 h combined inflammation and hypoxia stimulation (LPS/CC; 10 ng/mL LPS + 100 µmol/L CC) in 3T3-L1 adipocytes. Bars represent mean values ± SEM (*n* = 8–9/experimental group). Data were analyzed by one-way ANOVA followed by Tukey’s multiple comparison test. Bars not sharing a lower-case letter differ (*p* < 0.05).

The profile of inflammatory mediators secreted in response to combined inflammatory and hypoxic experimental conditions is shown in Figure 4. All SCFA and BCFA treatment groups exhibited reduced TNFα secretion compared to Con (*p* < 0.05); however, there were no differences between individual SCFAs or BCFAs. IsoBut + LPS/CC, IsoVal + LPS/CC, and Val + LPS/CC all increased secretion of IL-6, MCP-1/CCL2, MIP-1α/CCL3, and RANTES/CCL5 compared to both Con + LPS/CC and But + LPS/CC (Figure 4B,D,F,H; *p* < 0.05); however, there was no difference in the secretion of these same inflammatory mediators between Con + LPS/CC and But + LPS/CC (*p* > 0.05). Additionally, MIP-1β/CCL4 secretion was lower in But + LPS/CC compared to IsoBut + LPS/CC (Figure 4G; *p* < 0.05). When comparing SCFA, But + LPS/CC reduced secretion of IL-1β and MIP-1β/CCL4 compared to Val + LPS/CC (Figure 4C,G; *p* < 0.05). In contrast, the adipocyte secretory profile did not differ between Val + LPS/CC and IsoVal + LPS/CC or between BCFA (IsoBut + LPS/CC and IsoVal + LPS/CC) (*p* < 0.05), indicating that these two BCFA exert similar effects. Collectively, these data highlight the differences between comparable carbon chain length SCFA and BCFA (i.e., But versus IsoBut) and individual SCFA (i.e., But versus Val) under combined inflammation and hypoxic conditions and demonstrate how But is able to prevent the increase in inflammatory mediator secretion that was increased by the other SCFA and BCFA.

### 3.5. Effect of SCFA and BCFA on Transcription Factor Activtation in LPS/CC-Stimulated Adipocytes

**Figure 5:**
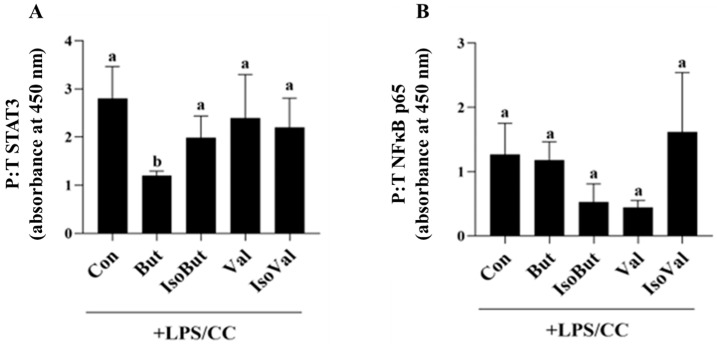
Effect of individual SCFA and BCFA (1 mmol/L) on transcription factor cellular protein expression (ratio of phosphorylated to total, P:T) of (**A**) STAT3, and (**B**) NFκB p65, in response to 24 h combined inflammatory and hypoxic (LPS/CC; 10 ng/mL LPS + 100 µmol/L CC) stimulation in 3T3-L1 adipocytes. Bars represent mean values ± SEM (*n* = 8–9/experimental group). Data were analyzed by one-way ANOVA followed by Tukey’s multiple comparison test. Bars not sharing a lower-case letter differ (*p* < 0.05).

In response to combined LPS + CC stimulation, only But reduced the ratio of phosphorylated to total STAT3 protein levels compared to Con and the other SCFA and BCFA treatment groups (*p* < 0.05; Figure 5A), that did not differ from each other. There was no difference between treatment groups in the ratio of phosphorylated to total NFκB p65 in response to LPS + CC stimulation (*p* > 0.05; Figure 5B).

## 4. Discussion

The current study demonstrated the differential effects of analogous carbon chain length SCFA (But and Val) and BCFA (isoBut and IsoVal) on adipocyte inflammatory mediator secretion, namely cytokines and chemokines, that represent types of adipokines that contribute to obese adipose tissue low-grade chronic inflammation, increase immune cell recruitment, and subsequent metabolic dysfunction [63]. The effects of individual SCFA and BCFA were assessed under unstimulated normoxic, inflammatory normoxic (i.e., LPS), and inflammatory hypoxic (i.e., LPS/CC) conditions. Adipocyte cultures were treated with SCFA and BCFA at the same concentration, 1 mmol/L, which has been used previously in adipocyte cultures assessing the metabolic effects of isobutyric acid and isovaleric acid on metabolic function [53], the effects of SCFA on metabolic function [33,36,39] and the effects of acetate [55] and acetate, propionate and butyrate on leptin and adiponectin levels [37]. Importantly, the concentration of SCFA used in this study is within the range of SCFA concentrations observed in human blood [64,65], wherein circulating SCFA concentrations can be variable depending on the underlying physiological condition [18,19,20,21,22,23]. Importantly, the SCFA and BCFA concentration levels used in this study have been shown previously to not affect adipocyte viability [39,53], which we also confirmed, wherein other studies assessing the metabolic effects of SCFA used higher concentrations that do not reflect circulating levels [34,44,45,46,55]. Conversely, little is known about the blood isovaleric acid and isobutyric acid concentrations, wherein circulating levels are lower in vivo [66,67], because microbial production of BCFA is typically lower compared to SCFA [48]. BCFA production and circulating levels reaching the extra-intestinal tissues, including adipose tissue, would be expected to increase with higher dietary intakes of protein and/or branched-chain amino acids (BCFA fermentation precursors) [68], a common supplementation strategy to support muscle development wherein excessive supplementation levels are associated with adverse outcomes such as hypertension [69,70]. For direct comparison between analogous carbon chain length SCFA and BCFA structures the same dose was utilized; however, future studies should include a dose-response to determine the minimal concentration of each microbial metabolite that is required to alter adipocyte inflammatory mediator secretion.

The nature of the stimuli used in the current study recapitulated the critical features of obese adipocytes, via an LPS concentration that mimics the circulating endotoxin levels reported in obese humans [56] and rodent high-fat diet-induced obesity models [57,58]. Further, LPS is a potent inflammatory stimulus signaling through toll-like receptor(TLR)-2 and TLR4 that results in the increased production of a profile of inflammatory mediators (namely cytokines and chemokines) that contribute to sustaining chronic low-grade inflammation and metabolic dysfunction in obesity [6,31,71]. LPS was used in combination with cobalt chloride, the chemical hypoxia mimetic that is not environmental hypoxia but has been shown to reproduce the effects of low (1%) oxygen tension between adipocytes without affecting cell viability [59,60]. Chronic low-grade inflammation in obesity can lead to adipose tissue hypoxia [6], which can further exacerbate adipocyte inflammation, the sustained secretion of adipokines such as inflammatory cytokines and chemokines that promote further immune cell recruitment into adipose tissue that become additional cellular sources (beyond adipocytes) of inflammatory mediators that collectively contribute to impaired adipogenesis, insulin resistance and metabolic dysfunction [63,72,73]. Furthermore, this connection between adipose tissue inflammatory mediator secretion and metabolic dysfunction is further exacerbated by hypoxia [10,63], and therefore, the combined inflammatory and hypoxic (LPS/CC) stimulation condition reflects a cell culture model of a more severe obese adipocyte phenotype [59,60]. It is important to assess how individual SCFA and BCFA may augment inflammatory mediator secretion in response to inflammatory normoxic and inflammatory hypoxic environmental conditions (i.e., LPS and/or LPS/CC, respectively), as the adipocyte response may differ with changes in the severity of the environmental conditions within obese adipose tissue. This is highly relevant when considering the translation of these results to humans, as the recommendations for the dietary intake of NDC and/or branched-chain amino acids that are required to support optimal microbial production of SCFA and BCFA, respectively may differ based on the physiology of the individual. The effects of individual SCFA and BCFA that may beneficially affect physiological functions in adipose tissue or other extra-intestinal tissues need to be identified, as a limitation of the current study was the use of a cell culture model with one cell type that demonstrates the effects of SCFA and BCFA on adipocytes but does not capture the cellular complexities of adipose tissue in vivo. Individual SCFA and BCFA cannot be expected to exert similar functions in extra-intestinal tissues, as demonstrated by the results in the current study and the effects of individual SCFA on skeletal muscle inflammatory mediator production, and insulin-stimulated glucose uptake [41,42], adipose tissue leptin secretion [44], and individual BCFA effects on IL-6 and lipid metabolism gene expression [52] and glucose uptake in adipocytes [53].

Metabolic effects of BCFA and SCFA in adipocytes or adipose tissue have been reported, whereas less is known about inflammatory adipokine production. Shorter chain BCFA (isobutyric acid and isovaleric acids) effects in adipocytes include decreased lipogenesis, lipolysis inhibition, and increased glucose uptake, wherein the dose required to elicit these effects differed between individual BCFA [53]. Similarly, in adipose tissue or adipocytes, SCFA have been reported to decrease lipolysis and increase triglyceride accumulation, glucose uptake, and adipogenesis [33,34,35,36,37,38,39]. Further, individual SCFA have been shown to have inconsistent effects on adipose tissue leptin and adiponectin gene expression or secretion [37,44,45]. Little is known about other adipokines, namely the cytokine and chemokine secretory profile, apart from a 3 mM propionic acid treatment on overweight human adipose tissue cultures [46]. At this higher dose than what was used in the current study, propionic acid reduced the adipose tissue secretion of chemokines (MIP-1α/CCL3, MIP-1β/CCL4, RANTES/CCL5, and CXCL10) and cytokines (TNFα, IL-4, and IL-10) [46]. In the current study, in the LPS-induced inflammatory condition, But + LPS had a strong anti-inflammatory effect compared to Con + LPS, including reduced secretion of a profile of mediators including IL-6, IL-1β, MCP-1/CCL2, MCP-3/CCL7, MIP-1α/CCL3, and RANTES/CCL5. Interestingly, the inflammatory mediator secretory profile differed between the SCFA, wherein Val + LPS increased secretion of IL-1β, MCP-1/CCL2, MIP-1α/CCL3, and RANTES/CCL5 compared to But + LPS. Comparable carbon chain length SCFA and BCFA also exhibited different adipocyte inflammatory mediator secretory profiles, apparent in But + LPS versus IsoBut + LPS secretion of IL-1β, MCP-1/CCL2, MCP-3/CCL7, MIP-1α/CCL3, MIP-1β/CCL4, and RANTES/CCL5, despite a similar effect of reducing expression of phosphorylated to total STAT3 and NFκB p65, the transcription factors that drive the expression of inflammatory mediators in response to LPS [71]. Conversely, the secretion profile did not differ between Val + LPS and IsoVal + LPS or the BCFA IsoBut + LPS and IsoVal + LPS. These findings demonstrate that individual SCFA are not physiologically equivalent, and therefore, identifying the effects of individual SCFA is needed, particularly within the context of the adipocyte microenvironment (i.e., LPS stimulation under normoxic versus hypoxic conditions). Collectively, these data demonstrate a potent non-inflammatory effect of But during LPS stimulation that is not attributable to the same extent to another individual SCFA, Val, or their analogous carbon chain length BCFA (IsoBut and IsoVal, respectively).

The assessment of inflammatory adipokine secretion in response to hypoxic inflammatory conditions was conducted, as hypoxic adipose tissue triggers the secretion of inflammatory adipokines that cause metabolic dysfunction [5,10]. In this connection, HIF-1α expression in adipocytes plays an important role in mediating overall adipose tissue inflammation [74]. Many chemokines are downstream transcriptional targets of HIF-1α that increase the recruitment of additional immune cells into adipose tissue; in particular, M1 macrophages that play a key role in metabolic dysfunction in obesity, including insulin resistance and glucose intolerance [5,10]. In the current study, *Hif1a* mRNA expression was reduced by But + LPS/CC compared to all other treatment groups, that did not differ from each other. Conversely, mRNA expression of *Angtl4*, the hypoxia-sensitive gene that has also been shown to affect adipocyte metabolic function including angiogenesis, lipid metabolism, and glucose homeostasis [62] was decreased by both IsoBut + LPS/CC and IsoVal + LPS/CC. In response to the combined inflammatory and hypoxic adipocyte microenvironment, But + LPS/CC reduced only TNFα secretion compared to Con + LPS/CC and the expression of phosphorylated to total STAT3. Otherwise, these two experimental groups exhibited a similar inflammatory mediator secretory profile. In contrast, the secretory profile for many inflammatory mediators was increased by the other SCFA (Val + LPS/CC) and BCFA (IsoBut + LPS/CC and IsoVal + LPS/CC) experimental groups compared to Con + LPS/CC, indicative of a proinflammatory effect. Val + LPS/CC was more proinflammatory compared to But + LPS/CC, and increased secretion of IL-6, IL-1β, MCP-1/CCL2, MIP-1α/CCL3, MIP-1β/CCL4 and RANTES/CCL5. Conversely, there were no differences in inflammatory mediator secretion between the two BCFA, IsoBut + LPS/CC and IsoVal + LPS/CC. In contrast, a comparison between analogous carbon chain length SCFA and BCFA highlighted their differential effects. There were no differences between Val + LPS/CC and IsoVal + LPS/CC, however, compared to IsoBut + LPS/CC, secretion of IL-6, MCP-1/CCL2, MIP-1α/CCL3, and RANTES/CCL5 was reduced by But + LPS/CC. Therefore, in comparison to Con, But did not exert the suppressive effect on inflammatory mediator secretion under hypoxic inflammatory conditions that was observed in response to LPS alone, importantly, But + LPS/CC sustained secretion levels relative to Con + LPS/CC and prevented a further increase in the inflammatory secretory profile that was observed in the other experimental groups. This could be interpreted as a beneficial outcome and a protective effect in response to But during inflammatory hypoxia. Collectively, these data highlight that individual SCFA and BCFA have unique effects on adipocyte inflammatory mediator secretion in response to different physiologic challenges associated with obesity (i.e., normoxic LPS-induced inflammation and hypoxic LPS-induced inflammation), and therefore, if these outcomes were extrapolated to humans, the beneficial effects of individual SCFA and BCFA would be dependent upon the severity of the obese adipose tissue microenvironment.

Collectively, the results from the current study highlight the differential effects of individual SCFA and their analogous carbon chain length BCFA counterparts the adipocyte adipokine/inflammatory mediator secretory profile under physiologically relevant inflammatory normoxic and inflammatory hypoxic experimental conditions. The findings show that the inflammatory mediator profile is differentially affected by each individual SCFA (i.e., But versus Val) and their corresponding analogous carbon chain length BCFA counterparts (i.e., But versus IsoBut, or Val versus IsoVal). This highlights the importance of assessing the individual effects of different SCFA and BCFA, without assuming that they will function similarly. Moreover, the differential responses of individual SCFA and BCFA under inflammatory normoxic and inflammatory hypoxic experimental conditions that recapitulate critical features of the cellular microenvironment that obese adipocytes would encounter [56,57,58,59], demonstrates that the adipocyte response to either SCFA or BCFA signals may differ depending on the severity and critical features of the adipocyte cellular microenvironment. Future studies should address (i) a comprehensive list of SCFA and BCFA (beyond those investigated herein); (ii) conduct a dose-response assessment to identify the minimal adipose tissue concentration required to elicit a beneficial effect to better inform dietary intake recommendations for precursors (NDCs and branched-chain amino acids); (iii) assess uptake and/or the signaling mechanisms through which each SCFA and BCFA elicits an effect on the inflammatory mediator secretory profile; and (iv) consider a profile of metabolic outcomes to better understand mechanistically how these microbial metabolites can potentially affect host adipocyte physiological function.

## 5. Conclusions

Comparable length SCFA and BCFA exert differential effects on the adipocyte secretory profile, wherein But reduces the release of inflammatory mediators under normoxic and hypoxic conditions, whereas IsoBut, Val, and IsoVal contribute to inflammation under hypoxic conditions.

## Figures and Tables

**Table 1:** Primer sequences.

Gene	Forward Primer (5′-3′)	Reverse Primer (5′-3′)
*Rplpo*	ACTGGTCTAGGACCCGAGAAG	TCCCACCTTGTCTCCAGTCT
*Hif1a*	AGGCTGGGAAAAGTTAGGAGTG	GGCAGCGATGACACAGAAAC
*Angptl4*	AGAAAACATGGGCTCGAGGG	TGGGAACCACAGTTAGCACC

## Data Availability

The original contributions presented in the study are included in the article, further inquiries can be directed to the corresponding author.
